# Targeting inflammation in glioblastoma: An updated review from pathophysiology to novel therapeutic approaches

**DOI:** 10.1097/MD.0000000000038245

**Published:** 2024-05-24

**Authors:** Nasser M. Alorfi, Ahmed M. Ashour, Adnan S. Alharbi, Fahad S. Alshehri

**Affiliations:** a Pharmacology and Toxicology Department, College of Pharmacy, Umm Al-Qura University, Makkah, Saudi Arabia; b Pharmacy Practice Department, College of Pharmacy, Umm Al-Qura University, Makkah, Saudi Arabia.

**Keywords:** anti-inflammatory, glioblastoma, inflammation, therapeutics, tumors

## Abstract

Glioblastoma (GBM) is a highly aggressive primary malignant brain tumor with a dismal prognosis despite current treatment strategies. Inflammation plays an essential role in GBM pathophysiology, contributing to tumor growth, invasion, immunosuppression, and angiogenesis. As a result, pharmacological intervention with anti-inflammatory drugs has been used as a potential approach for the management of GBM. To provide an overview of the current understanding of GBM pathophysiology, potential therapeutic applications of anti-inflammatory drugs in GBM, conventional treatments of glioblastoma and emerging therapeutic approaches currently under investigation. A narrative review was carried out, scanning publications from 2000 to 2023 on PubMed and Google Scholar. The search was not guided by a set research question or a specific search method but rather focused on the area of interest. Conventional treatments such as surgery, radiotherapy, and chemotherapy have shown some benefits, but their effectiveness is limited by various factors such as tumor heterogeneity and resistance.

## 1. Introduction

Glioblastoma (GBM) is a type of malignant brain tumor that arises from glial cells and the most common and aggressive form of primary brain tumors, accounting for approximately 15% of all brain tumors and 50% of all gliomas.^[1–3]^ This disorder is classified as a grade IV astrocytoma according to the World Health Organization classification system.^[4]^ It is characterized by its diffuse and infiltrative growth pattern, making it difficult to completely remove through surgery only.^[5]^ The exact cause of GBM is still not fully understood. However, several risk factors have been identified, including age, genetic mutations, exposure to ionizing radiation, and certain genetic disorders.^[6]^

Inflammation plays a complicated role in the development and progression of GBM.^[7]^ It can promote tumor growth, invasion, angiogenesis, and therapy resistance through various mechanisms, including immune cell activation, genetic and epigenetic alterations, and changes in the tumor microenvironment.^[8–10]^ Symptoms of GBM vary can include headaches, seizures, cognitive impairment, and weakness on one side of the body.^[11,12]^

GBM patients have almost 15-month median survival time despite years of research and clinical trials, despite decades of research and clinical trials.^[1,13]^ Genetic mutations and signaling pathways associated with GBM growth and survival have led to the development of targeted therapies. Similarly, the use of immunotherapy, such as immune checkpoint inhibitors and chimeric antigen receptor T-cell therapy, has shown promise in activating the immune system to recognize and attack GBM cells.^[14,15]^ Furthermore, recent clinical trials have evaluated the efficacy of combination therapies that involve different modalities, such as surgery, radiotherapy, and chemotherapy, as well as newer therapies like immunotherapy and gene therapy.^[16]^

### 1.1. Role of inflammation in GBM

One key mechanism by which inflammation influences GBM is through the activation of immune cells in the brain, such as microglia and macrophages.^[17]^ These immune cells can release pro-inflammatory cytokines and other molecules that promote tumor growth, invasion, and angiogenesis.^[18]^ Additionally, these immune cells can promote the development of an immunosuppressive tumor microenvironment, which delays the immune system’s ability to mount an effective antitumor response.^[19]^ Inflammation also affects the genetic and epigenetic alterations in glioblastoma cells.^[20]^ Studies have shown that inflammatory signaling pathways can activate oncogenic pathways and lead to DNA damage and mutations in glioblastoma cells, contributing to tumor initiation and progression.^[21,22]^ Studies have shown that inflammatory signaling pathways can be activated in response to chemotherapy and radiation, leading to treatment resistance and tumor recurrence.^[23,24]^ Inflammation-induced changes in the tumor microenvironment, such as increased angiogenesis and immunosuppression, can also promote resistance to therapy.^[25,26]^

## 2. Pathophysiology of glioblastoma

The most common subtype of GBM, known as primary GBM, arises de novo without any preceding low-grade precursor lesion.^[27,28]^ Secondary GBM arises from the transformation of a lower-grade astrocytoma.^[29]^ GBM is characterized by a wide range of genetic and epigenetic alterations that contribute to its aggressive behavior.^[30]^ These alterations include mutations in oncogenes, tumor suppressor genes, and genes involved in DNA repair and signaling pathways.^[14]^ Some of the most frequently mutated genes in GBM include TP53, PTEN, EGFR, IDH1, and NF1.^[31]^ One of the most important markers of GBM is its ability to infiltrate surrounding brain tissue, making complete surgical removal of the tumor impossible.^[32]^ This infiltrative growth pattern is due, in part, to the presence of glioma stem cells, which are a subpopulation of GBM cells that have the ability to self-renew and differentiate into multiple cell types.^[32,33]^ Glioma stem cells are thought to contribute to tumor initiation, maintenance, and resistance to therapy.^[34]^

## 3. Diagnosis of glioblastoma

Clinical diagnosis of GBM typically involves a thorough neurological examination, including assessments of motor and sensory function, reflexes, and coordination.^[35,36]^ Imaging studies, such as magnetic resonance imaging (MRI) and computed tomography, are also used to detect the presence of a brain tumor and to evaluate its location, size, and invasion into adjacent structures.^[37]^ MRI is the imaging modality of choice for diagnosing GBM due to its superior sensitivity and specificity in detecting brain tumors.^[38]^ The imaging features of GBM include a heterogeneously enhancing mass with irregular borders and central necrosis.^[39]^ Other imaging findings may include peritumoral edema, midline shift, and hydrocephalus.^[40]^ Histopathological examination of a biopsy or resected specimen is required to confirm the diagnosis of GBM and to determine its molecular subtype.^[41]^

Laboratory tests, such as blood tests and cerebrospinal fluid analysis, can also provide valuable diagnostic information in GBM.^[42]^ Blood tests can assess the levels of certain biomarkers, such as the glial fibrillary acidic protein, which is a marker of astrocytic differentiation and is commonly expressed in GBM.^[43]^ Cerebrospinal fluid analysis can detect the presence of cancer cells and can be useful in diagnosing leptomeningeal metastasis, which is a rare but serious complication of GBM.^[44]^ Genetic testing is also an important component of the diagnostic workup in GBM.^[45]^ Molecular profiling of the tumor can identify specific genetic alterations and mutations that can guide treatment decisions and predict prognosis.^[45]^

## 4. Pharmacological intervention

Treatments with anti-inflammatory drugs has emerged as a promising strategy for GBM management. Several classes of anti-inflammatory drugs have been studied for their potential efficacy against GBM, including nonsteroidal anti-inflammatory drugs (NSAIDs), corticosteroids, and immune modulators.

*NSAIDs*: NSAIDs are a class of drugs that inhibit the activity of cyclooxygenase (COX) enzymes, which are involved in the production of pro-inflammatory prostaglandins.^[46,47]^ Preclinical studies have shown that NSAIDs can inhibit GBM cell proliferation, induce apoptosis, and reduce angiogenesis.^[48]^ One of the key mechanisms by which NSAIDs may exert antitumor effects in GBM is through the inhibition of COX-2, an isoform of the COX enzyme that is upregulated in GBM cells.^[49]^ COX-2 expression has been shown to be associated with increased tumor aggressiveness and poorer prognosis in GBM patients.^[49]^ Selective COX-2 inhibitors, such as celecoxib, can inhibit GBM cell proliferation, induce apoptosis, and reduce angiogenesis. Celecoxib has also been shown to inhibit the invasion of GBM cells through the downregulation of matrix metalloproteinases, which are enzymes involved in the breakdown of extracellular matrix, a crucial step in tumor invasion.^[50]^ NSAIDs may also modulate the inflammatory microenvironment in GBM. Inflammatory mediators released by GBM cells contribute to the establishment of an immunosuppressive microenvironment that promotes tumor growth and invasion.^[51]^ NSAIDs have been shown to reduce the production of pro-inflammatory cytokines, chemokines, and prostaglandins, and inhibit the activation of immune cells, such as tumor-associated macrophages and microglia, which play a role in supporting tumor growth and immune evasion in GBM.^[46]^ NSAIDs may also interact with other signaling pathways involved in GBM pathogenesis. For example, NSAIDs have been shown to inhibit the Akt/mTOR pathway, which is frequently dysregulated in GBM and plays a role in cell survival, proliferation, and angiogenesis.^[52]^ NSAIDs have also been shown to modulate the Wnt/β-catenin pathway, which is involved in GBM stem cell self-renewal and differentiation.^[53]^ These findings suggest that NSAIDs may have pleiotropic effects on multiple pathways involved in GBM development and progression.

*Corticosteroids*: Corticosteroids are commonly used in the management of glioblastoma to alleviate symptoms related to tumor-induced edema and inflammation.^[54]^ The use of corticosteroids in GBM is primarily aimed at reducing peritumoral edema, which can lead to symptoms such as headache, nausea, and neurological deficits.^[55]^ Corticosteroids, such as dexamethasone and prednisone, exert their effects by reducing inflammation and edema through various mechanisms.^[56,57]^ They act by inhibiting the production of inflammatory mediators, such as prostaglandins and cytokines, and reducing capillary permeability, thus reducing the accumulation of fluid in the peritumoral tissue.^[55]^ Corticosteroids also have immunosuppressive effects, which can further help in reducing inflammation and edema in GBM.^[58]^

Prolonged use of corticosteroids can lead to various adverse effects, including hyperglycemia, immunosuppression, osteoporosis, muscle wasting, and gastrointestinal bleeding.^[59]^ It may also have potential negative effects on tumor growth, as they can inhibit the immune response against tumor cells and promote angiogenesis, which is a critical process in tumor progression.^[60]^ It can also interfere with the assessment of treatment response and monitoring of disease progression, as they can mask the clinical and radiological changes in the tumor. Furthermore, studies investigating the impact of corticosteroids on overall survival and disease progression in GBM have yielded conflicting results. Some review studies have suggested that prolonged corticosteroid use may be associated with lower survival outcomes in GBM patients,^[61]^ while others have not found a significant association. The optimal duration and dosage of corticosteroid therapy in GBM remain unclear, and the potential risks and benefits need to be carefully weighed in each individual patient.^[54]^ Overall, corticosteroids play a role in the management of GBM by reducing peritumoral edema and alleviating symptoms.

## 5. Conventional treatments for glioblastoma

### 5.1. Surgery techniques and outcomes

Conventional surgical techniques, such as craniotomy, are commonly used to remove the tumor and obtain a biopsy for histopathological analysis.^[62]^ The goals of surgery in GBM include maximizing the extent of resection, reducing mass effect, and relieving symptoms.^[63]^ However, the extent of resection is often limited by the infiltrative nature of the tumor and the proximity of critical brain structures, such as the motor and language areas.^[64]^ Several advanced surgical techniques have been developed to improve the outcomes of surgery in GBM. These techniques include fluorescence-guided surgery, intraoperative magnetic resonance imaging, and awake craniotomy.^[65]^ Fluorescence-guided surgery involves the use of fluorescent dyes, such as 5-aminolevulinic acid, which are selectively taken up by tumor cells and can be visualized under a specialized microscope.^[66]^ This technique can improve the extent of removal and reduce the risk of tumor recurrence.^[67]^ Intraoperative magnetic resonance imaging involves the use of a specialized MRI machine that is located in the operating room, allowing for real-time imaging during surgery.^[68]^

The outcomes of surgery in GBM depend on several factors, including the extent of resection, the location and size of the tumor, and the patient’s overall health. Despite advances in surgical techniques, GBM remains a challenging tumor to treat, and the majority of patients experience tumor recurrence and disease progression.^[63,69]^

### 5.2. Radiotherapy techniques and outcomes

Radiotherapy is the standard treatment approach for GBM and is typically delivered after surgery to target residual tumor cells and prevent recurrence.^[70]^ Conventional radiotherapy techniques, such as external beam radiation therapy, are commonly used in the treatment of GBM.^[71]^ External beam radiation therapy involves the delivery of high-energy radiation to the tumor site from an external source.^[72]^ The radiation is delivered in small daily fractions over a period of several weeks, to maximize tumor control while minimizing radiation-related toxicity to surrounding normal tissues.^[73]^ The outcomes of radiotherapy in GBM depend on several factors, including the total radiation dose, the fractionation schedule, and the patient’s overall health.^[74]^ In general, higher radiation doses are associated with better tumor control and survival outcomes. However, higher doses also increase the risk of radiation-related toxicity, such as radiation necrosis, which can lead to neurological deficits.

Advanced radiotherapy techniques, such as intensity-modulated radiation therapy and stereotactic radiosurgery, have been developed to improve the outcomes of radiotherapy in GBM.^[75,76]^ IMRT allows for the delivery of higher radiation doses to the tumor while sparing normal tissues, thereby reducing the risk of radiation-related toxicity.^[77]^ SRS involves the delivery of a high dose of radiation to the tumor in a single fraction, using a highly precise targeting system.^[78]^ The outcomes of advanced radiotherapy techniques in GBM have been promising, with some studies showing improved tumor control and survival outcomes compared to conventional radiotherapy techniques. However, these techniques are not without risks, and careful patient selection and monitoring are essential to minimize the risk of radiation-related toxicity

### 5.3. Chemotherapy drugs and outcomes

Chemotherapy is an important component of the multimodal treatment approach for GBM and is typically administered in combination with surgery and radiotherapy.^[79]^ Chemotherapy drugs work by targeting rapidly dividing cancer cells and preventing them from growing and dividing.^[75]^ The most commonly used include temozolomide (TMZ) and carmustine (BCNU).^[80]^ Newer chemotherapy drugs, for example, bevacizumab and temsirolimus, have been investigated in clinical trials for the GBM management.^[81]^

#### 5.3.1. Temozolomide

TMZ is an alkylating chemotherapy drug that is commonly used in the treatment of GBM.^[82]^ It is an oral drug has shown efficacy in improving survival outcomes in patients with GBM.^[83]^ TMZ has a high oral bioavailability and is rapidly absorbed in the gastrointestinal tract.^[84]^ The elimination half-life of TMZ is approximately 1.8 hours, and the drug is generally administered daily for several weeks in combination with radiotherapy after surgery.^[85]^ TMZ works by alkylating DNA and inducing cytotoxicity in rapidly dividing cancer cells. The drug is converted into a reactive intermediate that methylates the O6 position of guanine, which leads to the formation of DNA adducts and crosslinks, ultimately resulting in DNA damage and cell death.^[86]^

#### 5.3.2. Carmustine

BCNU is an alkylating chemotherapy drug that has been used in the treatment of GBM for several decades.^[87]^ It is an intravenous drug that has shown efficacy in improving survival outcomes in patients with GBM.^[88]^ BCNU is a lipophilic drug that readily crosses the blood-brain barrier. It is rapidly metabolized in the liver by the cytochrome P450 enzyme system and has a half-life of approximately 15 to 20 hours.^[89]^ The drug is generally administered every 6 to 8 weeks after surgery. BCNU works by alkylating DNA and inducing cytotoxicity in rapidly dividing cancer cells.^[90]^ The drug is converted into a reactive intermediate that reacts with DNA to form adducts and crosslinks, ultimately resulting in DNA damage and cell death.^[88]^

BCNU is associated with several side effects, including myelosuppression, gastrointestinal toxicity, and pulmonary toxicity.^[90]^ Myelosuppression is the most common side effect and can result in thrombocytopenia, leukopenia, and anemia.^[91]^ Gastrointestinal toxicity can result in nausea, vomiting, and diarrhea, which can be managed with antiemetic medications and supportive care measures. Pulmonary toxicity, which is less common, can result in interstitial pneumonitis and fibrosis and can be life-threatening.^[92]^ One notable side effect of BCNU is the development of delayed neurotoxicity, which can occur several months after treatment.^[93]^ This can result in progressive cerebellar ataxia, dementia, and other neurological symptoms.^[93]^

#### 5.3.3. Bevacizumab

Bevacizumab is a monoclonal antibody has been used in the treatment of GBM.^[94]^ It is an intravenous drug that has shown promise in improving progression-free survival in patients with GBM.^[95]^ Bevacizumab has a half-life of almost 20 days and is administered intravenously every 2 to 3 weeks.^[96]^ The drug does not cross the blood-brain barrier, so it works by blocking vascular endothelial growth factor signaling in the periphery and disrupting angiogenesis in the tumor microenvironment.^[97]^ Bevacizumab works by binding to and inhibiting vascular endothelial growth factor, thereby blocking the growth of new blood vessels and reducing the blood supply to the tumor.^[98]^ This leads to tumor cell death and improved clinical outcomes in some patients.

Bevacizumab is associated with several side effects, including hypertension, bleeding, thromboembolism, gastrointestinal perforation, and impaired wound healing.^[96]^ Hypertension is the most common side effect and can be managed with antihypertensive medications.^[99]^ Bleeding and thromboembolism are less common but can be life-threatening.^[100]^ Gastrointestinal perforation is a rare but serious complication that can lead to abdominal pain, fever, and sepsis.^[101]^

#### 5.3.4. Temsirolimus

Temsirolimus is an intravenous drug that belongs to the class of mammalian target of rapamycin (mTOR) inhibitors and has been investigated for its use in the treatment of GBM.^[102]^ Temsirolimus has a half-life of approximately 17 to 30 hours and is administered intravenously once weekly.^[103]^ mTOR is a key regulator of cellular growth and survival pathways, and it is frequently dysregulated in cancer, including GBM.^[104]^ Temsirolimus works by inhibiting mTOR, thereby blocking downstream signaling pathways that promote tumor cell growth and survival.^[105]^

Temsirolimus is associated with several side effects, including hematologic toxicity (neutropenia, anemia, thrombocytopenia), hyperglycemia, hyperlipidemia, rash, and mucositis.^[103,105]^ Hematologic toxicity is the most common side effect and can be managed with dose reductions or supportive care.^[106]^ Hyperglycemia and hyperlipidemia are common metabolic side effects and can be managed with lifestyle modifications or medication.^[107]^ Rash and mucositis are less common but can be managed with supportive care or topical treatments.^[108]^

## 6. Emerging therapeutic approaches for glioblastoma

Immunotherapy has emerged as a promising treatment strategy for GBM, including the use of checkpoint inhibitors, chimeric antigen receptor T-cells, and vaccines. Checkpoint inhibitors are monoclonal antibodies that target inhibitory receptors on T cells, such as PD-1 and CTLA-4, which can be upregulated in the tumor microenvironment and inhibit the antitumor immune response.^[109,110]^

## 7. Conclusion

Recent updates in GBM treatment represent important advancements in our understanding of this devastating disease and offer new opportunities for improving patient outcomes. Further efforts and collaborations are needed from researchers, clinicians, and patients to continue making progress in the fight against GBM.

## Author contributions

**Conceptualization:** Nasser M. Alorfi.

**Data curation:** Nasser M. Alorfi, Fahad S. Alshehri.

**Formal analysis:** Nasser M. Alorfi.

**Funding acquisition:** Nasser M. Alorfi.

**Investigation:** Nasser M. Alorfi.

**Methodology:** Nasser M. Alorfi.

**Validation:** Adnan S. Alharbi.

**Visualization:** Ahmed M. Ashour.
